# Multidimensional Assessment of COVID-19-Related Fears (MAC-RF): A Theory-Based Instrument for the Assessment of Clinically Relevant Fears During Pandemics

**DOI:** 10.3389/fpsyt.2020.00748

**Published:** 2020-07-31

**Authors:** Adriano Schimmenti, Vladan Starcevic, Alessandro Giardina, Yasser Khazaal, Joël Billieux

**Affiliations:** ^1^ Faculty of Human and Social Sciences, UKE—Kore University of Enna, Enna, Italy; ^2^ Faculty of Medicine and Health, Sydney Medical School, Nepean Clinical School, Discipline of Psychiatry, University of Sydney, Sydney, NSW, Australia; ^3^ Institute of Psychology, University of Lausanne, Lausanne, Switzerland; ^4^ Lausanne University Hospital and Lausanne University, Lausanne, Switzerland; ^5^ Department of Psychiatry and Addiction, Université de Montréal, Montréal, QC, Canada

**Keywords:** coronavirus disease 2019, fear, psychopathology, assessment, Multidimensional Assessment of COVID-19_Related Fears

## Abstract

In this article, we present the development and psychometric properties of the Multidimensional Assessment of COVID-19--Related Fears (MAC-RF). The MAC-RF is an eight-item, self-report scale that has been developed to assess clinically relevant domains of fear during the COVID-19 pandemic. The MAC-RF is based on a comprehensive theoretical model conceptualizing fears during the pandemics as resulting from an interaction of bodily, interpersonal, cognitive, and behavioral experiences. The MAC-RF was administered to a sample of 623 Italian adults from the community aged between 18 and 76 years old (M= 35.67, SD= 12.93), along with a measure of current clinical symptoms. Item response theory analyses demonstrated that each item of the MAC-RF provided sufficient information about the underlying construct of fear. The statistical fit of the scale was satisfactory. MAC-RF total scores correlated significantly and positively with total scores on the measure of psychopathology and with the clinical symptom domain scores. A ROC (receiver operating characteristic) curve analysis showed that the MAC-RF total score was sufficiently able to identify cases with high levels of current psychopathology, with an area under the curve of.76. These findings suggest that the MAC-RF can be used to assess pathological fear during pandemics. The English, Italian, and French versions of the MAC-RF are annexed to this article for use by clinicians and health services.

## Introduction

Fear is an unpleasant emotion caused by the perception of threat, which relates to danger, harm, or pain. This emotion stems from subcortical and cortical interactions that especially involve the “affective network” system of the brain (1), which includes the amygdala, orbitofrontal cortex, temporal cortex, pallidum, and insular cortex, among other structures. The amygdala and the thalamic pathways are responsible for the automatic and rapid appraisal of threat, whereas the hippocampus and the cortical pathways provide more detailed information on the specific context and characteristics of the threatening stimuli (2). Thus, activation of the amygdala by threatening stimuli is cognitively processed by the prefrontal and orbitofrontal cortex, leading to an experience of fear.

Fear emerged during brain evolution to allow animals to cope with dangers, e.g., by escaping or freezing (3), and it is usually activated by potentially dangerous external stimuli that evoke stress responses modulated by the hypothalamic-pituitary-adrenal (HPA) axis and glucocorticoid hormones. The relationship between fear and stress is complex and although both are often experienced concurrently, stress is assumed to be broader and usually encompassing fear. Therefore, stress responses do not always entail fear, while acute fear typically occurs as a stress response (4).

High levels of fear in humans represent a threat to the sense of safety and security, which elicit further negative emotions and generate alterations in physiological arousal and reactivity (5), distress, and heightened anxiety sensitivity (6). These alterations increase the risk of emotion dysregulation (7) and consequently, the risk of psychopathology (8). In fact, intense experiences of fear, especially when prolonged in time, may alter the regulation of genes controlling the neuroendocrine response to stress (e.g., by an excessive synthesis and secretion of glucocorticoids) (9), fostering physical and mental diseases (10).

Notably, fear experiences are common during a pandemic. Pandemics are unique in terms of causing prominent fear responses because the infection is transmitted invisibly, rapidly and with an increased risk of mortality (11). This limits the capacity of individuals to use adequate emotion regulation strategies (e.g., positive reappraisal of the situation) for coping with the situation, which has often been the case during the COVID-19 pandemic. It has been suggested that the pandemic has generated intense fear experiences among many individuals (12), that an adequate screening of these fears is necessary (13, 14) and that in some cases psychological interventions are needed (15). Therefore, understanding fears in the context of the COVID-19 pandemic is important, using both a theoretical model and a valid measure that would assess the fears and test the model.

### The “Four Horsemen” of Fear

Schimmenti, Billieux, and Starcevic (12) proposed a theoretical framework to account for fear experiences during the COVID-19 pandemic. They argued that a pandemic might generate fears that involve main psychological means of grasping the reality. Accordingly, bodily, relational, cognitive, and behavioral domains are involved in fear experiences during a pandemic, with these four domains of fear being interrelated. Furthermore, the model proposes that the four domains of fear are not structured in a hierarchical manner, and that instead, they are organized around a dialectical structure: the bodily domain involves a) fear of the body and b) fear for the body; the interpersonal domain involves c) fear of others and d) fear for others; the cognitive domain involves e) fear of knowing and f) fear of not knowing; the behavioral domain involves g) fear of action and h) fear of inaction. **Table 1** summarizes the types, characteristics and domains of fear experience according to Schimmenti et al.’s (12) theoretical model.

**Table 1:** Fear experiences during the coronavirus pandemic.

Types of fear experience	Characteristics of fear experience	Fear domains
*Fear of the body*	Sense of one’s physical vulnerability due to which the body is perceived as a potential source of danger.	Bodily domain
*Fear for the body*	Notion that one’s body needs to be protected.	
*Fear of others*	Threat originates from contacts with other persons, including key attachment figures, because of the possibility of being infected.	Interpersonal domain
*Fear for others*	Threat concerns one’s contacts with other people, including the loved ones, because of the possibility of infecting them.	
*Fear of knowing*	Avoidance of information about the pandemic as a way of reducing the effect of the perceived threat.	Cognitive domain
*Fear of not knowing*	Coping with negative feelings by collecting all information about the pandemic.	
*Fear of action*	Indecisiveness about taking action and a sense of being paralyzed by uncertainty.	Behavioral domain
*Fear of inaction*	Inner pressure to take action and do anything to avoid negative feelings and thinking about the pandemic.	

This theoretical model of fear experiences might contribute to our understanding of the origin and maintenance of fear-related symptoms during the COVID-19 pandemic. Based on the described theoretical framework, we developed a brief instrument, the Multidimensional Assessment of COVID-19-Related Fears (MAC-RF). The name of the measure reflects the fact that it is aimed to assess the four dimensions of fear described in the theoretical model.

The MAC-RF could be used for several purposes. First, the MAC-RF could serve as a screening instrument for clinically significant fears, which would indicate a need for a thorough clinical assessment if screening positive. Second, the MAC-RF could be used to identify the specific fear experiences that would help tailor preventive and treatment approaches. Third, this tool could serve the purpose of monitoring changes in the level of fear over time and measuring treatment response. This article aims to present the development and preliminary psychometric properties of the MAC-RF.

## Method

### Development of the Scale

Before starting the study, we first established the quality criteria for assessing the adequacy of the MAC-RF as follows:

- The measure should assess all eight facets of fear identified in Schimmenti et al.’s (12) theoretical model;
- The measure should be brief, easy to administer and easy to score to facilitate its use in health and community services, i.e., only one item for each of the eight fear facets should be retained;
- The measure should have satisfactory psychometric properties in terms of internal structure and convergent and predictive validity (i.e., the scale should be positively, significantly, and at least moderately correlated with an independent measure of psychopathology and it should be able to identify individuals with clinically significant fear experiences associated with psychopathology).

In order to develop a measure that would be consistent with the quality criterion (a), the first author developed an original pool of 16 items to correspond to each facet of fear (two items for each type of fear experience described in **Table 1**). Each item was scored on a 5-point Likert scale (from 0 = very unlike me to 4 = very like me), with higher scores on each item indicating higher levels of the corresponding fear facet. The wording of all 16 items was discussed with members of the research team and it was iteratively modified until consensus was reached on face validity of each item. It was then decided that an instrument with all 16 items would be administered in the validation study, with its final version including only a single item for each facet of fear, in accordance with the quality criterion (b).

### Participants

Participants in this study were 623 Italian community-dwelling adults (448 females, 71.9%) recruited online, ranging in age from 18 to 76 years (M= 35.67, SD= 12.93). Participants had 16.52 years of education on average (SD= 3.18). Their employment status was as follows: employed (n=231, 37.1%), self-employed (n=149, 23.9%), full-time students (n=151, 24.2%), homemakers (n=20, 3.2%) and unemployed (n=72, 11.6%). Only 9% of participants (n=56) lived alone and 8.7% (n=54) lived with friends, whereas the majority lived with their partner and offspring (n= 265, 42.5%) or with parents (n= 248, 39.8%). At the time of the survey completion, the mean duration of pandemic-related restrictions, such as lockdown, self-isolation, or quarantine, was 48.82 days (SD=12.47).

### Procedure

The study received an approval from the institutional review board for psychological research of the first author’s university (code UKE-IRBPSY-04.20.04). Participation was anonymous and voluntary and participants received no compensation for completing the survey. Participants were recruited by advertisements placed on social media platforms, with a request for the survey to be disseminated *via* respondents’ social media platforms. Participants signed an electronic informed consent before being directed to an online survey, which consisted of a sociodemographic questionnaire, the pool of 16 items developed by the research team to assess fear-related experiences during the pandemic according to Schimmenti et al.’s (12) theoretical model, a measure of psychopathology, and additional instruments not directly related to the objective of the current study. The survey was opened for 10 days, from 27 April 2020 to 5 May 2020. Of 628 total respondents, 623 completed the survey. The survey software did not allow participants to skip any question and therefore, there were no missing or incomplete responses in the dataset.

### Measures

#### Sociodemographic Questionnaire

Participants were asked to provide sociodemographic information, including gender, age, number of years of education, employment status, and marital status during the COVID-19 pandemic.

#### Multidimensional Assessment of COVID-19-Related Fears

The MAC-RF is an eight-item self-report measure scored on a five-point Likert scale (from 0 to 4) that was developed by the authors of this article to assess the eight facets of fear identified by Schimmenti et al. (12). The MAC-RF was derived from a set of 16 items. Using item response theory (IRT) analysis, the eight items that were most discriminating for each facet of fear (i.e., those displaying the higher *a*-value in the current study) were selected and included in the final version of the instrument. Scores of the MAC-RF can range from 0 to 32, with higher scores indicating higher COVID-19-related fears. The MAC-RF was developed in Italian, French, and English languages (see the **Supplementary Material**), with team consensus on translation and back-translation of its items. Findings reported in this study concern the Italian version of the measure. The psychometric properties of the French and English version of the MAC-RF are still under examination, as data collection in French- and English-speaking countries started later than in Italy, in which lockdown measures have been taken since early March 2020. The psychometric properties of the Italian translation of the measure are extensively described below in the *Results* section.

#### DSM-5 Self-Rated Level 1 Cross-Cutting Symptom Measure-Adult (CCSM)

The Cross-Cutting Symptom Measure-Adult (CCSM) is a 23-item, self-report measure used for screening of various domains of psychopathology. It assesses relevant clinical symptoms that occurred in the preceding 2 weeks on a 0 to 4 Likert scale (from “none” to “severe”). The CCSM is included in the Diagnostic and Statistical Manual of Mental Disorders—Fifth Edition (DSM-5; American Psychiatric Association, 2013) and provides 13 clinical symptom domain scores (depression, anxiety, anger, mania, somatic symptoms, suicidal ideation, psychosis, sleep problems, memory problems, obsessive-compulsive symptoms, dissociation, maladaptive personality functioning, and substance use). A total score on the CCSM is obtained by averaging the scores on clinical symptom domains. A sample item is “little interest or pleasure in doing things” (related to the symptom domain of depression). The CCSM has demonstrated adequate psychometric properties in the DSM field trials (16) and many studies across the world (17, 18). Internal consistency (Cronbach’s alpha value) of the CCSM in the current study was.89.

### Statistical Analyses

To select the eight items to be retained in the final version of the MAC-RF, the Pearson’s *r* correlation between the item scores and the total score of the original sixteen items were examined. This procedure was complemented with an exploratory use of item response theory (IRT) analysis, and the *a*-parameter value of each item was calculated. For each pair of items per fear facet, we retained the item showing the highest correlation with the total score of the 16 items (thus the item being more consistent with the entire measure) and showing the highest *a*-value (thus showing the highest capacity to discriminate the hypothesized latent construct of COVID-19 related fear). Subsequently, we tested *via* exploratory factor analysis if the eight selected items would tap into a single factor. After verifying that this condition was met, unidimensional IRT analyses based on graded model were conducted to examine the psychometric properties of the final eight-item version of the MAC-RF in reflecting adequately the latent construct of COVID-19-related fears. In particular, we considered the values of the *a* parameter (the larger this value, the better the item is able to discriminate between people with varying degrees of the latent construct θ) and *b* parameter (where high *b* values indicate a diﬃcult item, that is, a decreased probability that high scores on the item are endorsed). We also examined the test information function (the amount of information yielded by the test at any level of the dimensionally conceptualized construct), and we assessed the goodness of fit of the IRT model by testing exact (M_2_) and approximate (root mean square error of approximation, RMSEA) fit. Nonsignificant M_2_ probability indicates exact fit. However, in IRT applications it is highly unlikely that a model will exactly fit the data, thus statistics for approximate fit are used, such as the RMSEA, that takes into account both the M_2_ value and the degrees of freedom of the model. An RMSEA below.05 indicates adequate fit, that is, indicates that the latent trait dimensionality is correctly specified (19). Descriptive statistics were then computed for all study variables. Gender differences were tested through t-tests for independent samples. Correlational analyses were performed to examine the associations between MAC-RF scores and scores on various domains of psychopathology.

A receiver operating characteristic curve (ROC) analysis was conducted to further test the ability of the MAC-RF to predict the severity of clinical symptoms in the sample, using the 75^th^ percentile of the CCSM total score to dichotomize between cases and non-cases with high levels of psychopathology (20, 21). By applying this rule of thumb, we were able to identify a cutoff value for the CCSM that took into account the global increase in clinical symptoms observed in the population as a response to the COVID-19 pandemic (22). Sensitivity (the proportion of true positive individuals with the condition in a total group of subjects with the condition), specificity (the proportion of participants without the condition with negative test result in the total of participants without the condition), positive likelihood ratio (the likelihood that positive test results occur in participants with the condition compared to those without the condition), negative likelihood ratio (the ratio of the probability that negative results occur in participants with the condition to the probability that the same result will occur in participants without the condition), positive predictive value (the probability of having the condition in a subject with positive results), and negative predictive value (the probability of not having the condition in participants with negative test result) were calculated to test the ability of the MAC-RF to identify participants with a “condition” (i.e., those with high levels of psychopathology).

## Results

### Item Selection, Internal Structure, and Reliability

Item-total correlation values and *a-*values of an IRT analysis based on graded model were first examined to select the single item to retain for each of the eight fear facets of Schimmenti et al.’s (12) theoretical model. **Supplementary Table 1** displays the original 16 items, their item-total correlation values, their *a-*values, and the outcome (whether or not they were retained for the eight-item MAC-RF). We compared the *r*s of item-total correlations and the *a*-values of the item pairs related to each fear facet, and then selected one item that better reflected each fear facet. This empirically based selection of items allowed us to include in the MAC-RF only one item for each fear facet, as per our quality criteria.

We then performed an exploratory factor analysis on the eight item of the MAC-RF, to test if a single-factor solution was tenable. We used the principal axis factoring method selecting the oblimin rotation to allow the potentially identified factors to correlate, as per theoretical model prediction. The data were homoscedastic [Bartlett’s χ^2^ (28) = 1,719.29, p <.001], and the sample size was adequate for factor analysis (Keyser-Meyer-Olkin = .87). A single factor was extracted that explained 41.47% of variance, with all items loading above.40 on the factor. The examination of the eigenvalues and the scree-plot clearly supported the single factor solution for the eight items (with the first five eigenvalues being 3.85,.91,.84,.67, and.54).

After the positive testing for unidimensionality, we proceeded with unidimensional IRT analysis of the final measure. The results of IRT analysis based on graded model are summarized in **Table 2**. Each item of the MAC-RF provided sufficient (item 5) to excellent (item 2) level of information on the latent construct of the specific fear facet. The most discriminant item (i.e., the item with higher *a-*value) was item 2 (concerning a fear for the body), while the most difficult item (i.e., the item with the highest *b*-value and with the lowest probability to receive a high score) was item 6 (related to the fear of not knowing).

**Table 2:** Multidimensional Assessment of COVID-19-Related Fears (MAC-RF) item parameter estimates.

Item	Facet of fear	*a*	*b*_1_	*b*_2_	*b*_3_	*b*_4_
1	Fear of the body	1.87	−0.37	0.43	1.15	1.89
2	Fear for the body	3.24	−0.46	0.19	0.79	1.44
3	Fear of others	2.03	−1.03	−0.05	0.78	1.86
4	Fear for others	2.11	−1.35	−0.49	0.18	1.00
5	Fear of knowing	0.93	−0.50	0.63	1.76	3.35
6	Fear of not knowing	1.20	−0.04	1.08	2.07	3.49
7	Fear of action	2.10	−0.14	0.62	1.24	2.05
8	Fear of inaction	1.11	−0.70	0.32	1.25	2.51

With regards to the information provided by the MAC-RF at different levels of θ (in IRT analyses, θ represents the latent variable that is standardized to have a mean of 0 and a standard deviation of 1), the MAC-RF provided most information on the latent construct of fear at levels of θ between 0 and + 0.8 (**Table 3**). The MAC-RF was not particularly informative at its lowest total scores, as expected of a measure that aims to identify individuals with clinically relevant levels of fear. With the MAC-RF total scores of 11 (which corresponds to a θ of 0 in the population-based distribution conversion table from summed score to scale score) or above, the instrument provided a highly relevant information on the latent construct of each fear facet. A total MAC-RF score of 20 corresponded to a θ of 1, suggesting that this score might be a cut-off value for identifying heightened experiences of fear that deserve clinical attention.

**Table 3:** Item information function of the Multidimensional Assessment of COVID-19-Related Fears (MAC-RF) at different values of θ (from −2.8 to 2.8).

Item	Facet of fear	−2.8	−2.4	−2.0	−1.6	−1.2	−0.8	−0.4	0.0	0.4	0.8	1.2	1.6	2.0	2.4	2.8
1	Fear of the body	0.04	0.07	0.15	0.29	0.51	0.77	0.97	1.06	1.09	1.09	1.09	1.05	0.94	0.72	0.46
2	Fear for the body	0.01	0.02	0.07	0.25	0.81	2.00	2.91	3.04	3.10	3.10	2.99	2.54	1.27	0.43	0.12
3	Fear of others	0.11	0.23	0.44	0.76	1.05	1.18	1.21	1.24	1.25	1.23	1.18	1.16	1.06	0.78	0.47
4	Fear for others	0.19	0.39	0.72	1.08	1.27	1.33	1.37	1.37	1.35	1.29	1.11	0.77	0.43	0.21	0.09
5	Fear of knowing	0.08	0.11	0.14	0.17	0.20	0.23	0.25	0.26	0.27	0.27	0.27	0.27	0.27	0.26	0.26
6	Fear of not knowing	0.05	0.08	0.11	0.17	0.23	0.30	0.37	0.41	0.44	0.45	0.46	0.46	0.45	0.44	0.44
7	Fear of action	0.02	0.04	0.08	0.19	0.39	0.71	1.07	1.28	1.35	1.37	1.36	1.33	1.24	1.00	0.63
8	Fear of inaction	0.10	0.14	0.19	0.25	0.30	0.34	0.37	0.39	0.39	0.39	0.39	0.38	0.37	0.36	0.32
Test information:		1.59	2.08	2.92	4.15	5.77	7.86	9.52	10.06	10.23	10.20	9.86	8.96	7.04	5.20	3.79
Expected s.e.:		0.79	0.69	0.59	0.49	0.42	0.36	0.32	0.32	0.31	0.31	0.32	0.33	0.38	0.44	0.51

A marginal reliability for response pattern scores was .87. **Table 4** shows factor loadings of the MAC-RF items. The statistics based on one-way, two-way, and full marginal tables showed a significant M_2_ (728.16, df=440, p<.001), but a satisfactory RMSEA of.03 indicating that the latent trait dimensionality was correctly specified in the IRT model. All eight items loaded positively and moderately to highly on the latent construct.

**Table 4:** Factor loadings of the Multidimensional Assessment of COVID-19-Related Fears (MAC-RF) items.

Item	Item content	*λ*	*s.e.*
1	I don’t trust my own body to protect me against the coronavirus infection.	0.74	0.05
2	I am frightened about my body being in contact with objects contaminated by the coronavirus.	0.89	0.03
3	I fear that people who are around me can infect me.	0.77	0.04
4	I am frightened about my family members or close friends being in contact with other people and becoming infected with the coronavirus.	0.78	0.04
5	I do not want to be exposed to information about the coronavirus infection because it makes me feel upset and anxious.	0.48	0.07
6	I feel upset if I cannot collect all the information I need about the coronavirus.	0.58	0.06
7	During the coronavirus pandemic I feel paralyzed by indecisiveness or fear of doing something wrong.	0.78	0.04
8	During the coronavirus pandemic I constantly feel that I have to do something.	0.55	0.06

Further analyses based on the classical test theory showed a good internal consistency (Cronbach’s alpha = .84) of the MAC-RF, satisfactory split-half reliability (Spearman-Brown r= .78), and an average inter-item correlation of.39 (thus in the suggested range between.20 and.40). All items of the MAC-RF were moderately to strongly correlated with its total score (from r = .54 to r = .80, all ps<.001).

These results suggest that the MAC-RF is an informative and reliable measure of COVID-19-related fears.

### Descriptive Statistics

MAC-RF total scores ranged from 0 to 30 (M=11.21, SD=7.04; interquartile range= 6–16; skewness=.36, kurtosis=−.68). CCSM total scores ranged from 0 to 3.51 (M=.96, SD=.62). **Table 4** displays descriptive statistics for the MAC-RF and CCSM scores for the full sample and for males and females separately.

As **Table 5** shows, the fear for significant others (item 4) was more strongly endorsed compared to all other fear facets. Participants also reported significant levels of symptoms of anger, depression, anxiety, mania, and sleep problems on average (more than one or two episodes in the preceding 2 weeks). Concerning gender differences, females reported significantly increased COVID-19-related fear experiences on all the items of the MAC-RF except for item 6, related to the fear of not knowing. As a result, MAC-RF total scores were also significantly higher in females. This pattern of results on the MAC-RF corresponds to the CCSM scores, where females reported significantly more symptoms than males. A series of t-tests for independent samples with Bonferroni’s correction for multiple comparisons showed that females reported significantly higher levels of the symptoms of depression, mania and anxiety, as well as somatic symptoms and memory problems, whereas males reported significantly higher levels of substance use.

**Table 5:** Descriptive statistics of the Multidimensional Assessment of COVID-19-Related Fears (MAC-RF) and Cross-Cutting Symptom Measure-Adult (CCSM) and gender differences.

	Total (N = 623)		Males (n = 175)		Females (n = 448)		t(621)	p
	M	SD	M	SD	M	SD		
MAC-RF total score	11.21	7.04	9.47	6.25	11.89	7.22	−3.91	<.001
MAC-RF item 1	1.27	1.31	0.96	1.11	1.39	1.36	−3.70	<.001
MAC-RF item 2	1.44	1.34	1.13	1.24	1.56	1.36	−3.62	<.001
MAC-RF item 3	1.65	1.23	1.45	1.16	1.72	1.25	−2.47	.014
MAC-RF item 4	2.20	1.37	1.87	1.26	2.33	1.39	−3.86	<.001
MAC-RF item 5	1.23	1.27	0.99	1.16	1.32	1.29	−2.99	.003
MAC-RF item 6	0.91	1.11	0.96	1.11	0.90	1.11	.66	.51
MAC-RF item 7	1.09	1.25	0.89	1.1	1.16	1.30	−2.45	.015
MAC-RF item 8	1.43	1.33	1.22	1.21	1.51	1.37	−2.46	.014
CCSM total score	0.96	0.62	0.85	0.58	1.00	0.63	−2.79	.005
Depression	1.64	1.07	1.42	1.07	1.73	1.06	−3.33	.001
Anger	1.91	1.20	1.78	1.24	1.96	1.19	−1.72	.085
Mania	1.32	0.99	1.20	0.93	1.37	1.01	−1.97	.050
Anxiety	1.47	1.05	1.08	0.96	1.63	1.05	−6.04	<.001
Somatic symptoms	0.90	1.03	0.71	0.92	0.97	1.07	−2.92	.004
Suicidal ideation	0.17	0.59	0.20	0.65	0.16	0.56	.71	.480
Psychosis	0.11	0.43	0.10	0.36	0.12	0.45	−.50	.621
Sleep problems	1.45	1.37	1.31	1.33	1.50	1.38	−1.54	.124
Memory problems	0.51	0.97	0.37	0.81	0.56	1.02	−2.26	.024
Obsession/compulsion	0.66	0.97	0.63	0.95	0.67	0.98	−.40	.693
Dissociation	0.57	1.00	0.45	0.89	0.62	1.03	−1.95	.052
Maladaptive personality	1.12	1.15	1.05	1.05	1.15	1.18	−1.01	.313
Substance use	0.62	0.80	0.74	0.82	0.57	0.79	2.42	.016

### Association With Psychopathology and Convergent Validity

The MAC-RF total and item scores were significantly correlated with CCSM total scores, with the levels of associations being in the moderate range (*r* = .55 for the association between MAC-RF total scores and CCSM total scores, *r*s ranging from.31 to.47 for the associations between MAC-RF item scores and CCSM total scores, all *p*s <.001). **Table 6** shows correlations between the MAC-RF total and item scores and the CCSM domain scores. All correlations between the total MAC-RF scores and CCSM domain scores were significant, except for substance use. MAC-RF total and item scores showed the strongest associations with anxiety symptoms. The patterns of these associations did not change when partial correlations were examined and the effects of gender, age, education, and days spent in pandemic-related restriction conditions were partialled out. Overall, the correlational findings support the convergent validity of the MAC-RF.

**Table 6:** Pearson’s r correlation between Multidimensional Assessment of COVID-19-Related Fears (MAC-RF) scores and Cross-Cutting Symptom Measure-Adult (CCSM) domain scores.

	Depression	Anger	Mania	Anxiety	Somatic symptoms	Suicidal ideation	Psychosis	Sleep problems	Memory problems	Obsession/compulsion	Dissociation	Maladaptive personality	Substance use
MAC-RF total score	.42**	.38**	.27**	.63**	.50**	.16**	.23**	.33**	.26**	.36**	.33**	.37**	.05
MAC-RF item 1	.35**	.25**	.10**	.47**	.40**	.11**	.16**	.22**	.15**	.24**	.20**	.24**	.08
MAC-RF item 2	.28**	.27**	.16**	.48**	.36**	.08*	.18**	.20**	.16**	.26**	.24**	.23**	−.02
MAC-RF item 3	.25**	.24**	.15**	.41**	.33**	.08*	.09*	.24**	.18**	.16**	.18**	.24**	.04
MAC-RF item 4	.28**	.24**	.18**	.44**	.33**	.08*	.12**	.25**	.19**	.24**	.22**	.26**	.01
MAC-RF item 5	.27**	.25**	.23**	.42**	.30**	.09*	.14**	.20**	.07	.24**	.22**	.26**	−.02
MAC-RF item 6	.25**	.25**	.15**	.35**	.29**	.07	.08	.18**	.21**	.20**	.18**	.17**	.08
MAC-RF item 7	.34**	.31**	.20**	.53**	.40**	.21**	.27**	.21**	.25**	.34**	.30**	.36**	.04
MAC-RF item 8	.31**	.30**	.29**	.38**	.36**	.13**	.21**	.27**	.23**	.34**	.24**	.29**	.08

* p<.01, ** p<.01 (two tailed).

### Identifying Cases With High Levels of Current Psychopathology

Finally, we performed a ROC curve analysis to test the ability of the MAC-RF to identify cases with high levels of current psychopathology. The 75^th^ percentile of the total CCSM score (i.e., the last quartile) corresponding to scores above 1.32 was used to delineate participants with high levels of current psychopathology. An area under the curve was.76 (95% C.I.72–.81, p<.001), indicating that the MAC-RF total score is sufficiently able to identify cases with high levels of current psychopathology. Examining the potential cut-off scores of the MAC-RF, we found that a cut-off score of 12 seemed to suggest high levels of current psychopathology. This is based on the sensitivity of 75.80%, specificity of 62.45%, positive likelihood ratio of 2.02, negative likelihood ratio of 0.39, positive predictive value of 40.48%, and negative predictive value of 88.45%. These findings confirm the positive relationship between COVID-19-related fears and overall psychopathology.

## Discussion

This article examined the psychometric properties of the Italian version of the MAC-RF, a theory-based measure that was developed for the screening and assessment of clinically relevant fears during the COVID-19 pandemic. Even though the MAC-RF is not the first measure that was developed to assess COVID-19-related fears (13, 23), it might have some theoretical and clinical advantages over other dedicated instruments. The advantages of a theory-based measure include interpretability of item scores according to theory, testing the theory itself, and the possibility to combine theory with results of the assessment to guide clinical decision-making. Our findings suggest that the MAC-RF adequately taps all the eight domains of fear during the COVID-19 pandemic proposed by Schimmenti and colleagues (12). The instrument might also have some predictive value in identifying individuals at increased risk of current psychopathology during the COVID-19 pandemic.

The eight items of the MAC-RF identified by IRT analysis provided sufficient to excellent information on the latent construct of fear (see **Table 2**), with values of the *a* parameter ranging from 0.93 to 3.24. Notably, the highest value of the *a* parameter was found for item 2, related to the fear for the body, and thus to the fear of being contaminated by the virus. This finding is consistent with research (13) and theory (24) suggesting that the most prominent fear during the pandemics relates to the risk of illness and death. Results of the validation studies of the other two instruments developed to assess fear and related constructs in the context of the COVID-19 pandemic are also in agreement with our findings. Thus, one of the item of the fear of COVID-19 scale on which the participants had the highest scores assessed a fear of death resulting from COVID-19 (13). Similarly, a factor concerning danger and contamination fears extracted from the COVID Stress Scales accounted for most variance compared to other factors (23). In contrast, the lowest value of the *a* parameter (and thus the lowest discrimination ability for the underlying construct of fear) was observed for item 5 of the MAC-RF which regards the fear of knowing. This result suggests that knowledge about COVID-19 and the associated risks is perceived as promoting a sense of control, with the fear of not having that knowledge being most distressing.

The most difficult item (i.e., the item with the highest value in the IRT *b* parameter) of the MAC-RF was the fear of not knowing, seemingly opposite from the item concerning the fear of knowing. This apparently paradoxical result is consistent with the theory on COVID-19-related fears proposed by Schimmenti et al. (12), which posits the dialectical alternation of fears of knowing and not knowing during the pandemic. This finding may be explained by the reluctance of many people to know “too much” about the pandemic to avoid being overwhelmed by frightening information.

The fit indices of the MAC-RF were good (see **Table 4**), with all items loading above.45 on the latent construct and a good RMSEA of.03 (25). Also, internal reliability was good and the average inter-item correlations were in the suggested range between.20 and.40 (26). Thus, the MAC-RF can be considered an internally valid and reliable measure of the fears related to COVID-19.

We also found gender differences, with females displaying higher levels of fears than males on the total MAC-RF scores and on all item scores, except for item 6 related to the fear of not knowing (see **Table 5**). These gender differences were analogous to gender differences on the general psychopathology scores, where females reported higher levels of depression, mania, anxiety, somatic symptoms, and memory problems than males, while males reported higher substance use than females. These findings are highly consistent with previous research reporting increased levels of fear among females (27, 28), and more generally with research showing that females are more prone to internalizing symptoms, such as anxiety and depression, whereas males are more prone to externalizing symptoms, such as substance use and antisocial behaviors (29, 30).

It is worth noting that the scores on various domains of psychopathology were quite high in our sample (see **Table 5**), with mean scores of 1 or above on the specific domains of depression, anger, mania, anxiety, sleep problems, and maladaptive personality functioning. This means that, on average, our participants reported the presence of symptoms related to these domains as occurring at least once in the 2 weeks before completing the survey. We believe that these high scores are a consequence of the pandemic situation. It has been suggested in recent literature that the COVID-19 pandemic may have profoundly negative effects on the overall functioning of individuals by altering their habits and daily life (31), evoking uncertainty and insecurity in the relationship between the self and the world (1) and causing intense anxiety responses (13).

Results of correlational analyses supported the convergent validity of the MAC-RF, whose items correlated positively and significantly with the total score of a measure assessing different types of psychopathological symptoms (see **Table 6**). This suggests that the MAC-RF assesses clinically relevant fears associated with a more severe current psychopathology. Notably, the strongest correlation of the MAC-RF was observed with anxiety, which is consistent with theory and neurobiological evidence that fear and anxiety are highly connected and that they overlap (2). However, the MAC-RF was positively and significantly associated with several other psychopathological domains, supporting a view that domains of fear assessed by the MAC-RF are relevant for identifying overall psychopathology and not only its anxiety domain.

We examined the ability of the MAC-RF to identify cases with high levels of current psychopathology *via* a ROC curve analysis. This analysis revealed that the MAC-RF performed sufficiently well in this regard, with an area under the curve of.76. However, the MAC-RF displayed an adequate sensitivity but a limited specificity at the suggested cut-off value of 12, indicating that its use in screening practice should be complemented with other specific measures on psychopathology. Nonetheless, the overall results of the ROC analysis, especially the positive likelihood ratio of 2.02 and the negative predictive value of 88.45%, suggests that the MAC-RF maintains some usefulness in identifying those individuals whose COVID-19-related fear experiences are associated with increased clinical symptoms.

The present study has several limitations. First, the MAC-RF is based on a specific theory about different domains of fear during a pandemic. While this is an advantage and the theory is rather comprehensive, it is possible that some relevant domains of fears have been overlooked by the theory and therefore, they are not assessed by the MAC-RF. Second, the study was cross-sectional, precluding any conclusions about possible causal relationships between domains of fear and various aspects of psychopathology. Longitudinal studies using the MAC-RF are needed to better understand these relationships. Third, findings of the study were based on self-report, which is subject to various biases. In this context, it is noteworthy that individuals with high levels of psychopathology have been identified using an empirically-derived cutoff value on a self-report measure. Future studies should test the validity of the MAC-RF against a more strict criterion, such as the presence of a psychiatric diagnosis. Finally, the study was conducted in Italian adults recruited online from the general population and its findings do not necessarily generalize to other population groups, such as adolescents, people with various mental disorders, and individuals from a different ethnic background. Therefore, studies in samples more clearly representative of the general population of various countries, as well as studies in clinical samples, are warranted.

## Conclusions

Findings of the present study support use of the MAC-RF as a brief, theory-based instrument for assessment of clinically relevant fears related to the COVID-19 pandemic. Although the MAC-RF was developed for use in the context of this pandemic, it could be administered to assess fear experience in other public health emergencies, especially future pandemics during which the causative agent spreads rapidly *via* human contact and is associated with mortality and much uncertainty. Modifications of the MAC-RF for this purpose would be simple, with changes in the wording of the relevant items (e.g., by replacing the term “coronavirus” with a term related to another pandemic situation). The MAC-RF was simultaneously developed in three languages and its versions in Italian, English, and French are presented in the **Supplementary Material** to this article.

## Data Availability Statement

The raw data supporting the conclusions of this article will be made available by the authors, without undue reservation.

## Ethics Statement 

The studies involving human participants were reviewed and approved by Internal Review Board for Psychological Research of the UKE - Kore University of Enna (code: UKE-IRBPSY-04.20.04). The participants provided their written informed consent to participate in this study.

## Author Contributions 

All authors contributed to the study design and to the development of the items of the Multidimensional Assessment of the COVID-19-Related Fears (MAC-RF). AS collected and analyzed the data, and was responsible for preparing the first draft of the article. All authors contributed to the article and approved the submitted version.

## Conflict of Interest

The authors declare that the research was conducted in the absence of any commercial or financial relationships that could be construed as a potential conflict of interest.

## Supplementary Material

Supplementary Table 1 Development of the MAC-RF: original items, related theoretical facets, item-total correlation, *a*-paramenter values, and retention of items.
