# Supplementary material for: Multidimensional Assessment of COVID-19-Related Fears (MAC-RF): A Theory-Based Instrument for the Assessment of Clinically Relevant Fears During Pandemics
Source: Front Psychiatry. 2020 Jul 31;11:748. doi: 10.3389/fpsyt.2020.00748 (PMC7411221; doi:10.3389/fpsyt.2020.00748)
Supplement: Supplementary Table 1 — Development of the MAC-RF: original items, related theoretical facets, item-total correlation, a-paramenter values, and retention of items. [file Table_1.docx]

Supplementary Table 1. Development of the MAC-RF: original items, related theoretical facets, item-total correlation, *a-*paramenter values, and retention of items.

| Item |  | Item-total correlation (*r*) | IRT *a-*parameter | Retained | Facet |
| --- | --- | --- | --- | --- | --- |
| 1 | I fear that I will have a severe form of illness in case of the coronavirus infection. | .66 | 1.64 | NO | Fear of the body |
| 9 | I don’t trust my own body to protect me against the coronavirus infection. | .69 | 1.85 | YES |  |
| 2 | I constantly look for signs of the coronavirus infection in my body. | .61 | 1.66 | NO | Fear for the body |
| 10 | I am frightened about my body being in contact with objects contaminated by the coronavirus. | .79 | 3.06 | YES |  |
| 3 | I fear that people who are around me can infect me. | .73 | 2.23 | YES | Fear of others |
| 11 | I want to maintain as much physical distance as possible because others can infect me. | .72 | 2.08 | NO |  |
| 4 | I fear that I have an undiagnosed coronavirus disease and for this reason I can infect people who are important in my life. | .66 | 1.56 | NO | Fear for others |
| 12 | I am frightened about my family members or close friends being in contact with other people and becoming infected with the coronavirus. | .74 | 2.21 | YES |  |
| 5 | I do not want to be exposed to information about the coronavirus infection because it makes me feel upset and anxious. | .52 | 0.93 | YES | Fear of knowing |
| 13 | I prefer to know as little as possible about the coronavirus infection. | .35 | 0.54 | NO |  |
| 6 | I feel upset if I cannot collect all the information I need about the coronavirus. | .57 | 1.26 | YES | Fear of not knowing |
| 14 | I have to know everything about the coronavirus to protect myself. | .48 | 0.93 | NO |  |
| 7 | During the coronavirus pandemic I feel paralysed by indecisiveness or fear of doing something wrong. | .72. | 1.98 | YES | Fear of action |
| 15 | I feel that during the coronavirus pandemic not taking any action may be the safest thing to do. | .47 | 0.91 | NO |  |
| 8 | During the coronavirus pandemic I constantly feel that I have to do something. | .56 | 1.05 | YES | Fear of inaction |
| 16 | During the coronavirus pandemic I feel some relief only if I take action. | .50 | 0.85 | NO |  |
