# Supplementary material for: Multidimensional Assessment of COVID-19-Related Fears (MAC-RF): A Theory-Based Instrument for the Assessment of Clinically Relevant Fears During Pandemics
Source: Front Psychiatry. 2020 Jul 31;11:748. doi: 10.3389/fpsyt.2020.00748 (PMC7411221; doi:10.3389/fpsyt.2020.00748)
Supplement: Supplementary file 2 [file DataSheet_1.docx]

**Supplementary Material**

**Multidimensional Assessment of COVID-19-Related Fears (MAC-RF)**

- ENGLISH -

(Adriano Schimmenti, Vladan Starcevic, Alessandro Giardina, Yasser Khazaal, Joël Billieux)

This is a list of things people might feel about the coronavirus. We are interested in how you would describe yourself. Please read each statement carefully and determine to what extent each statement may apply to you by selecting the number that corresponds the most to your experience during the past week.

|  | Very unlike me | Somewhat unlike me | Neither like or unlike me | Somewhat like me | Very like me |
| --- | --- | --- | --- | --- | --- |
| 1. I don’t trust my own body to protect me against the coronavirus infection. | 0 | 1 | 2 | 3 | 4 |
| 2. I am frightened about my body being in contact with objects contaminated by the coronavirus. | 0 | 1 | 2 | 3 | 4 |
| 3. I fear that people who are around me can infect me. | 0 | 1 | 2 | 3 | 4 |
| 4. I am frightened about my family members or close friends being in contact with other people and becoming infected with the coronavirus. | 0 | 1 | 2 | 3 | 4 |
| 5. I do not want to be exposed to information about the coronavirus infection because it makes me feel upset and anxious. | 0 | 1 | 2 | 3 | 4 |
| 6. I feel upset if I cannot collect all the information I need about the coronavirus. | 0 | 1 | 2 | 3 | 4 |
| 7. During the coronavirus pandemic I feel paralyzed by indecisiveness or fear of doing something wrong. | 0 | 1 | 2 | 3 | 4 |
| 8. During the coronavirus pandemic I constantly feel that I have to do something. | 0 | 1 | 2 | 3 | 4 |

**Multidimensional Assessment of COVID-19-Related Fears (MAC-RF)**

**-ITALIAN-**

(Adriano Schimmenti, Vladan Starcevic, Alessandro Giardina, Yasser Khazaal, Joël Billieux, 2020)

Di seguito è presentato un elenco di ciò che le persone potrebbero provare rispetto al coronavirus. A noi interessa il modo in cui descriveresti te stesso. Per favore, leggi attentamente ogni affermazione e stabilisci quanto ciascuna di esse corrisponde alla tua esperienza, selezionando il punteggio che corrisponde maggiormente a ciò che hai vissuto durante l’ultima settimana.

|  | Molto diverso  da me | Abbastanza diverso  da me | Nè diverso  da me nè simile a me | Abbastanza simile  a me | Molto simile  a me |
| --- | --- | --- | --- | --- | --- |
| 1. Non mi fido del fatto che il mio corpo possa proteggermi dall’infezione da coronavirus. | 0 | 1 | 2 | 3 | 4 |
| 2. Sono terrorizzato dal fatto che il mio corpo entri in contatto con oggetti contaminati dal coronavirus. | 0 | 1 | 2 | 3 | 4 |
| 3. Ho paura che le persone intorno a me possano contagiarmi. | 0 | 1 | 2 | 3 | 4 |
| 4. Sono terrorizzato dal fatto che i membri della mia famiglia o i miei amici stretti entrino in contatto con altre persone e vengano contagiate dal coronavirus. | 0 | 1 | 2 | 3 | 4 |
| 5. Non voglio essere esposto alle informazioni sull’infezione da coronavirus, perché ciò mi fa sentire a disagio e ansioso. | 0 | 1 | 2 | 3 | 4 |
| 6. Mi sento a disagio se non posso raccogliere tutte le informazioni di cui ho bisogno sul coronavirus. | 0 | 1 | 2 | 3 | 4 |
| 7. Durante la pandemia da coronavirus mi sento paralizzato dall’indecisione o dalla paura di fare qualcosa di sbagliato. | 0 | 1 | 2 | 3 | 4 |
| 8. Durante la pandemia da coronavirus ho costantemente la sensazione di dover fare qualcosa. | 0 | 1 | 2 | 3 | 4 |

| **Multidimensional Assessment of COVID-19-Related Fears (MAC-RF)**  **-FRENCH-**  (Adriano Schimmenti, Vladan Starcevic, Alessandro Giardina, Yasser Khazaal, Joël Billieux, 2020) | | | | | |
| --- | --- | --- | --- | --- | --- |
| Vous trouverez ci-dessous une liste de préoccupations que toute personne pourrait éprouver concernant le coronavirus. Nous aimerions savoir comment vous vous situez par rapport à ces dernières. Merci de bien vouloir lire attentivement chaque énoncé, et d’indiquer votre degré d’accord ou de désaccord selon votre expérience vécue au cours de la semaine passé. | | | | | |
|  | Tout à fait d'accord | Plutôt en accord | Ni en accord ni en désaccord | Plutôt en désaccord | Tout à fait en désaccord |
| 1. Je n’ai pas confiance dans la capacité de mon corps à résister à l’infection par le coronavirus | 0 | 1 | 2 | 3 | 4 |
| 2. Je suis effrayé(e) à l’idée que mon corps puisse être en contact avec des objets contaminés par le coronavirus. | 0 | 1 | 2 | 3 | 4 |
| 3. J’ai peur que mon entourage puisse m’infecter. | 0 | 1 | 2 | 3 | 4 |
| 4. Je suis effrayé(e) à l’idée que les membres de ma famille ou mes amis proches soient en contact avec d’autres personnes, et puissent se faire contaminer par le coronavirus. | 0 | 1 | 2 | 3 | 4 |
| 5. Je ne veux pas être confronté(e) aux information liées au coronavirus car cela me perturbe et me rend anxieux(se). | 0 | 1 | 2 | 3 | 4 |
| 6. Je suis contrarié(e) si je n’ai pas accès à toutes les informations dont j’ai besoin concernant le coronavirus. | 0 | 1 | 2 | 3 | 4 |
| 7. Pendant cette pandémie de coronavirus, je suis paralysé(e) par l’incertitude et la peur de faire quelque chose de mal. | 0 | 1 | 2 | 3 | 4 |
| 8. Pendant cette pandémie de coronavirus, j’ai constamment le sentiment de devoir être en train de faire quelque chose. | 0 | 1 | 2 | 3 | 4 |
